# Implementing online learning of Diagnostic Criteria for the Temporomandibular Disorders (DC/TMD) protocol in undergraduate dental education

**DOI:** 10.2340/aos.v83.40984

**Published:** 2024-06-26

**Authors:** Jaana Malmberg, Ritva Näpänkangas, Marjo-Riitta Liljeström, Johanna Tanner, Tuija Teerijoki-Oksa, Auli Suominen, Kirsi Sipilä

**Affiliations:** a Department of Oral and Maxillofacial Diseases, Turku University Hospital, Turku, Finland; b Department of Prosthetic Dentistry and Stomatognathic Physiology, Institute of Dentistry, University of Turku, Turku, Finland; c Research Unit of Population Health, Faculty of Medicine, University of Oulu, Oulu, Finland; d Medical Research Center, Oulu University Hospital and University of Oulu, Oulu, Finland; e Department of Community Dentistry, Institute of Dentistry, University of Turku, Turku, Finland

**Keywords:** DC/TMD, undergraduate dental education, online

## Abstract

**Introduction:**

The Diagnostic Criteria for Temporomandibular Disorders (DC/TMD) is part of the undergraduate dental curriculum. Online teaching has nowadays become common also in dentistry.

**Objective:**

To compare undergraduate students’ self-assessed ability and satisfaction with learning DC/TMD Axis I between traditional and online learning and to evaluate the possible gains of online teaching.

**Material and Methods:**

Third-year undergraduate dental students in 2018 (traditional learning, Group 1, *n* = 43/50) and in 2019 (online learning, Group 2, *n* = 34/50) at the University of Oulu, Finland evaluated their self-assessed ability and satisfaction with learning DC/TMD clinical examination and diagnostics on a 10-point scale. Additionally, those participating in online courses answered to two open-ended questions; Group 2 (*n* = 50) and another group from the University of Eastern Finland in 2019 and 2020 (*n* = 75, Group 3). Total of 105/125 students (84%) responded. Content analysis was used to open-ended responses.

**Results:**

The online course reported significantly higher self-assessed ability in measurements (*p* = 0.004), identifying referred pain (*p* = 0.043) and statement for the diagnostics (*p* = 0.017) and also higher self-assessed satisfaction in measurements (*p* = 0.046). According to the content analysis, essential gains of online teaching were efficient learning, videos and exercises, and adjustability to own timetable.

**Conclusion:**

The online learning course can be considered as a good option for traditional learning of the DC/TMD protocol.

## Introduction

The Diagnostic Criteria for Temporomandibular disorders (DC/TMD) have been developed by the International Network for Orofacial Pain and Related Disorders Methodology (INfORM; formerly the International RDC/TMD Consortium Network) in order to standardize the diagnostic criteria of most common temporomandibular disorders worldwide [1, 2]. The DC/TMD protocol consists of two axes: Axis I to define the physical diagnosis and Axis II for psychosocial assessment. The Axis I diagnosis is based on the DC/TMD Symptom Questionnaire, clinical examination, and diagnostic algorithms using the DC/TMD Decision tree and Diagnostic Criteria.

In many countries, temporomandibular disorders (TMD)/orofacial pain (OP) education is part of the dental school curriculum [3, 4]. The programme differs considerably between schools [5] as studies have found that dental students have not acquired sufficient skills in diagnosis and management of TMD/OP [3, 5].

In pedagogical literature, traditional learning is defined as a learning process where learners and experts are present physically in the same place at the same time, and the knowledge is transferred face to face, and no technological mediation of communication between teacher and students is required [6]. In traditional learning, the lecturer transmits knowledge to students [7].

Online learning has been described as a synchronous or asynchronous interaction in a web-based system between instructor(s)/expert(s) and students [8]. Online learning approach has become an attractive and very necessary method. It allows for the selection of the time and place of learning and often provides learners with interactive self-learning tools.

Compared to traditional learning, self-learning is assumed to be more effective in online environment, because it is stated to improve the quality of learning and increase the cost-effectiveness of education [9, 10]. When students’ experiences and learning in online and traditional learning were compared, both modalities lead to similar levels of academic performance, but students preferred written activities online and discussions in person [11].

Based on the INfORM Consortium guidelines, DC/TMD can be educated at three levels: self-learning, a 2-day didactic course and a 2-day didactic course with reliability assessment [12]. The education includes theoretical basis of the DC/TMD and clinical training of Axis I clinical examination protocol, where mandatory commands, measurements, and muscle and joint palpations are practised. The DC/TMD protocol has been a part of both undergraduate and postgraduate curricula in Finnish universities for several years as the Finnish translation was introduced in 2016. In the beginning, the learning method was traditional with face-to-face lectures following Axis I hands-on clinical training of examination protocol including diagnostics. Advances in online technologies and their increasing use in education have raised interest for alternative methods of education also in undergraduate education concerning the DC/TMD theoretical background in order to achieve readiness for hands-on practices. As there is limited data for online learning on DC/TMD Axis I protocol, more studies are needed to evaluate its applicability.

## Aim

The aim of the study was to evaluate the self-assessed ability and satisfaction to perform the DC/TMD Axis I hands-on training of clinical examination protocol including diagnostics, when comparing online learning with traditional learning. The aim was also to evaluate the possible gains of online teaching.

## Material and methods

### Participants

Three groups of third-year undergraduate dental students attended different courses on DC/TMD Axis I, using traditional or online learning methods:

1. 50 students (22 males, 28 females) at the University of Oulu, Finland, attending a traditional course in 2018 (Group 1).
2. 50 students (14 males, 36 females) at the University of Oulu, Finland, attending an online course in 2019 (Group 2).
3. 75 students (25 males, 50 females) at the University of Eastern Finland, attending an online course in 2019 and 2020 (Group 3).

All the students were fluent in Finnish.

The course on DC/TMD Axis I included theoretic basis and hands-on training of clinical examination protocol including diagnostics. The learning objectives of the courses for the participants were to achieve the ability to perform the clinical examination and diagnostics according to DC/TMD Axis I protocol, which have been translated in Finnish (DC/TMD-FIN) [13].

### Learning methods

The traditional learning consisted of four traditional lectures (introduction to the DC/TMD protocol, DC/TMD criteria, and clinical examination protocol (Axis I), performance of the clinical examination according to DC/TMD criteria, and diagnostics according to the DC/TMD protocol). The DC/TMD examination instructional video (in Finnish) was shown during a lecture concerning clinical examination performance according to the DC/TMD protocol.

The online learning consisted of online modules in a web-based Moodle learning platform. Modules included recorded video lectures of the same topics as in the traditional learning, the DC/TMD examination instructional video (in Finnish), additional online material related to DC/TMD, and exams for self-evaluation (embedded answers [Cloze] on required examination verbal commands in the DC/TMD protocol and multiple-choice questions concerning the Axis I diagnoses based on two patient cases).

After theoretical education (traditional or online), all the groups (Groups 1–3) performed the DC/TMD Axis I hands-on training protocol including diagnostics in groups of three students (one acting as patient, one acting as examiner and one as recorder, then changing the roles after the examination). Teachers followed the performance of the students and gave instructions and feedback, if needed.

### Questionnaire

**Figure 1:**
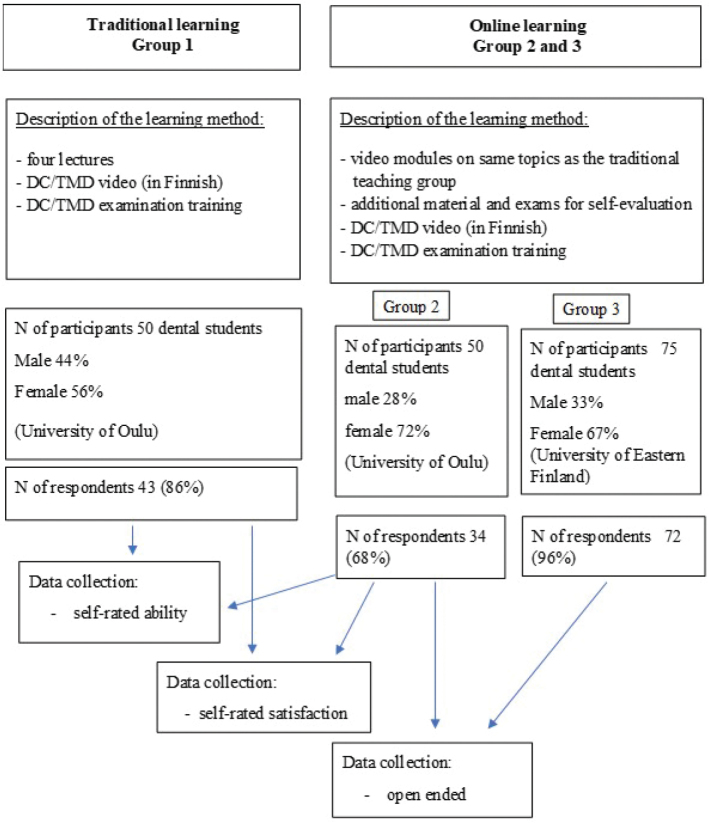
Description of the study design.

The study design is presented in Figure 1. The students of Group 1 and Group 2 were asked to fill in an anonymous self-evaluation questionnaire after the DC/TMD Axis I hands-on training of clinical examination protocol including diagnostics. The questionnaire, which contained 10 questions, was identical to the questionnaire of Vilanova et al., and it was translated in Finnish [14]. The reliability of the self-assessed ability and satisfaction questionnaire was good, the Cronbach’s alpha being 0.878 and 0.920, respectively. The questions, using a 10-point scale, inquired the ability to perform the clinical examination (0 = no ability, 10 = very high ability) and the satisfaction regarding learning in aspects of the DC/TMD clinical examination protocol (0 = not satisfied, 10 = very satisfied) as followed: Give correct instructions to the patient; Identify pain localization; Measurements (range of motion, overbite, etc.); Assessment of temporomandibular joint (TMJ) sounds; Palpation of muscles; Palpation of TMJ; Identifying familiar pain; Identifying referred pain; Derive the DC/TMD-diagnosis (Axis I) [14]. Forty-three students (86%) in the traditional learning course, and 34 students (68%) in the online learning course filled in the self-evaluation questionnaire.

### Qualitative evaluation of the online learning course

The online learning courses (Groups 2 and 3) were inquired to answer two open-ended questions. The questions inquired the essential gain from the course and development suggestions. The question inquiring essential gain of the course was written as follows: The most essential gain of the online course for me (name the three most essential issues). The responses to open-ended questions were analysed using inductive content analysis [15, 16]. Firstly, the open-ended answers were grouped by similarities into subthemes, which were further combined into two larger main themes by one of the writers (JM) (Table 1).

**Table 1:** Content analysis method (inductive) procedure used for the analysis of open-ended questions Groups 2 and 3.

Open ended question title	Original expression	Subtheme (*n* of answers)	Themes
Essential gain from the course.	*‘Course gave thorough knowledge of how to make diagnosis’.* *‘Learning how to use diagnosis tree, getting acquainted to subject and different disorders’.*	Learning DC/TMD clinical examination and diagnostics (*n* = 63)	DC/TMD clinical examination and diagnosis
	*‘Videos, which could be revisited, were good’*	Educational videos and adjustability of course to own timetables (*n* = 27)	
	*‘TMD diagnostics in practise’.*	Patient cases and exercises supporting learning (*n* = 22)	
	*‘Learning anatomics and discus dislocation dynamics’*	Recapitulation of anatomic features and understanding of disorders (*n* = 9)	
Development suggestions	*‘More clinical examples and practical aspects in the videos’. ‘More interactive action on lectures, possibly recapitulation of anatomic features’.*	Change/development of content or structure of teaching (*n* = 18)	Change/develop content or structure of teaching
	*‘Possibly more exercises (patient cases) to make it easier to adopt the content’.*	Development of more functional exercises (*n* = 16)	
	*‘It would be nice, if the exercises would work at the first time’*	Solving technical problems and making technic adjustments (*n* = 7)	
	*‘The course seemed to be a bit messy, as there were not, or I didn’t find the instructions’.*	Offering better instructions and material centralized into one place (*n* = 4)	

DC/TMD: Diagnostic Criteria for the Temporomandibular Disorders.

The students were provided with verbal information about the aims and details of the study, and they were asked to provide online consent to use their replies in this study. In Finland, no formal ethical review for this type of questionnaire-based study was required (Medical Research Act). However, ethical principles of the Helsinki Declaration were rigorously adhered to in the study. The participants responded to the questionnaire anonymously with no identification data and gave their consent for use of data. The participation in the research was voluntary. The data contained no identifiers, and the participants could not be recognized in the research reports.

### Statistical analysis

Response ratings of the questions were described using medians and interquartile ranges (IQRs). The differences between Groups 1 and 2 were tested using Mann–Whitney U test for not normally distributed sums of self-assessed ability and satisfaction. Data were processed with IBM SPSS Statistics (version 27) statistical software.

Ad hoc effect size and sample size calculations were done. The effect size of one-point ended at 74% power with the group size of 50 students. The effect size of one-point needed 58 students in each group with 80% power to be found statistically significant at 5% alpha.

The statistical significance was considered at *p* < 0.05. Analyses were conducted using IBM SPSS Statistics 27.

## Results

### Self-assessed ability

The medians and IQRs of self-assessed ability and the statistical significances of the differences between the traditional and online learning courses, Groups 1 and 2, are shown in Table 2. The overall highest medians with lowest variation were achieved in Group 1 in ‘Identifying familiar pain’ and in Group 2 in ‘Measurements’. For Group 1, the lowest scores were in ‘Statement for Diagnostics’. For Group 2, the lowest scores were in ‘Giving correct instructions to the patient’, ‘Palpation of muscles’ and ‘Statement for the Diagnostics’. In the comparison of Groups 1 and 2, statistically significant differences in favor of Group 2 were found in the self-assessed ability in ‘Measurements’ (*p* = 0.004), ‘Identifying referred pain’ (*p* = 0.043), and ‘Statement for the Diagnostics’ (*p* = 0.017) (Table 2).

**Table 2:** The self-assessed ability to perform the DC/TMD clinical examination (0 = no ability, 10 = very high ability) in undergraduate dental online and traditional courses (according to Vilanova et al. 2015), presented as median (IQR) and p-values for difference between courses.

Ability	Group 1 Traditional (*n* = 43)**	Group 2 Online (*n* = 34)*	Group 1 Traditional vs. Group 2 Online
	median (IQR)	median (IQR)	*p* ***
Give correct instructions to the patient	7 (3)	7 (3)	0.689
Identify pain localization	7 (2)	8 (2)	0.841
Measurements	8 (3)	9 (1)	0.004
Assessment of temporomandibular joint sounds	8 (2)	7 (2)	0.734
Palpation of muscles	8 (2)	7 (3)	0.231
Palpation of temporomandibular joints	8 (2)	8 (2)	0.509
Identifying familiar pain	8 (1)	8 (2)	0.328
Identifying referred pain	7 (3)	8 (2)	0.043
Statement for the Diagnostics	6 (2)	7 (3)	0.017

IQR: interquartile range.

* missing 4.

** missing 9.

*** Mann–Whitney U test.

### Self-assessed satisfaction

The medians and IQRs of respective different categories in self-assessed satisfaction and the differences between Groups 1 and 2 are shown in Table 3. The highest values with lowest variation were achieved in Group 1 in ‘Identifying familiar pain’ and in Group 2 in ‘Measurements’. For Group 1, the lowest scores were in ‘Statement for the diagnostics’ and for Group 2 in ‘Giving correct instructions to the patient’. In the comparison of Groups 1 and 2, Group 2 had statistically significantly higher self-assessed satisfaction compared to Group 1 in ‘Measurements’ (*p* = 0.046).

**Table 3:** The self-assessed satisfaction regarding learning of the DC/TMD clinical examination protocol (0 = not satisfied, 10 = very satisfied) in undergraduate dental online and traditional courses (according to Vilanova et al. 2015) presented as median (IQR) and p-values for difference between courses.

Satisfaction	Group 1 Traditional (*n* = 43)**	Group 2 Online (*n* = 34)*	Group 1 Traditional vs. Group 2 Online
	median (IQR)	median (IQR)	*p* ***
Give correct instructions to the patient	7 (2)	7 (3)	0.941
Identify pain localization	8 (2)	8 (3)	0.809
Measurements	8 (3)	8.5 (1)	0.046
Assessment of temporomandibular joint sounds	8 (2)	8 (3)	0.683
Palpation of muscles	8 (2)	7.5 (3)	0.867
Palpation of temporomandibular joints	8 (3)	8 (2)	0.532
Identifying familiar pain	8 (1)	8 (3)	0.553
Identifying referred pain	7 (3)	8 (3)	0.356
Statement for the Diagnostics	6 (4)	8 (2)	0.057

IQR: interquartile range.

* missing 4.

** missing 9.

*** Mann–Whitney U test.

### Open-ended questions for the online learning course (Groups 2 and 3)

The subthemes arising from the essential gain of online learning course category were ‘Learning DC/TMD examination and diagnostics’, ‘Educational videos and adjustability of the course to own timetables’, ‘Patient cases and exercises supporting learning’ and ‘Recapitulation of anatomic features and understanding the mechanism of disorders’ (Table 1). The most frequent theme arising from subthemes (more than 50% of the answers) in essential gain of the course was ‘Learning DC/TMD examination and diagnostics’.

The subthemes arising from development suggestions to the online learning course were as follows: ‘Change/develop content or structure of teaching’, ‘Develop more functional exercises’, ‘Solve technical problems and make technique adjustable’, and ‘Better instructions and material centralized to one place’ (Table 1). In development suggestions, the distribution of subthemes was more uniform, and the two most frequent themes arising from subthemes (more than 50% of the answers) were ‘Develop more applicable exercises’ and ‘Change or develop the content or structure of teaching’.

## Discussion

In the present study, online and traditional learning methods for DC/TMD teaching were evaluated in undergraduate dental education. Both the traditional and online courses were targeted to give readiness for the clinical practices, that is, examination and diagnostics of the patients based on the DC/TMD Axis I. Thus, both the courses ended up these examinations based on different ways of theoretical learning. The results showed that in measurements, identifying referred pain and statement for the diagnostics the students attending the online learning course assessed higher ability and satisfaction than those attending the traditional learning course in the theoretical teaching for performing the DC/TMD Axis I hands-on clinical training of examination protocol including diagnostics.

In the 21st century, a large variety of technologies are commonly utilised in education. Different learning environment terms overlap each other, which has created confusion and difficulties when performing meaningful cross-study comparisons. Online learning has been apparently the most difficult to define. For some authors, online learning is the complete online learning experience [17], and for others, it is just the technology medium or context with which it is used [18]. In this study, we used the term online learning, as online learning is described by most authors as access to learning experience via the use of technology [19–21].

The present study showed that in some aspects students assessed more self-ability and self-satisfaction on online teaching than on traditional method. Other studies have also resulted in promising results. The combined online and traditional learning method (blended learning) has already been successfully implemented in some universities to teach radiology, orthodontics, and stomatognathic physiology, for example [22–27]. In another study, it was found that online modules may be used successfully to improve undergraduate dental students’ perceptions of the basic sciences and enhance their ability to apply basic science content to clinically important scenarios [23]. In yet another study, a virtual learning environment (VLE) platform providing information about the TMJ, TMD and teaching of a thorough TMJ examination was developed and compared with conventional teaching by a cross-over trial. The findings indicated that no differences were found between teaching modes, and both modes were equally effective at delivering information to students [26]. In addition, both augmented reality (AR) and virtual reality (VR) have been tested in education [28, 29], also in dental education [30].

In the present study, the difference between the online and traditional learning was found in students’ self-assessed ability to perform measurements (range of motions, overbite etc.), identifying referred pain and statement for the diagnostics in favor of the online learning course. The evaluations differed between these courses also in the students’ self-assessed satisfaction in measurements, again in favor of online learning. The explanation for this could be the possibility to watch online lectures and instructional video as many times as required to understand the content. The easy access, time, and place flexibility as well as activating exercises may also favor the online learning course to traditional face-to-face learning [31]. In contrast to the traditional course, the online course also included additional material and exams for self-evaluation, and therefore this difference may have caused benefit for the online course students. Although creating an online learning course demands lots of resources in the beginning, afterwards it enables redirection of teachers’ resources to other aspects of teaching and saves time.

Based on the content analyses of the open-ended questions, the core learning outcome was achieved as the students found the most important gain from the course to be learning DC/TMD clinical examination and diagnostics. The suggestions for development mostly concerned the structure of the course and the exercises (for example patient cases). In addition, the function of techniques is very important for an online learning course to succeed, as mentioned in the answers. Although the younger generation uses technology and different applications without problems, in a recent study, there was a notable decline in confidence in completing learning tasks among community college students when due to Covid-19 they had to shift to technology-based remote learning without prior online course experience [7]. These findings seem to underline the fact that shifting to online learning needs planning and proper implementation and participants’ perceptions should be monitored, studied, and improvements made, if needed. The freedom from time and place, however, makes an online teaching course a tempting alternative for self-instructed learning, and this could be utilized in the DC/TMD Axis I protocol teaching as well. Performing the structural DC/TMD clinical protocol correctly and accordingly achieving the correct diagnosis is the most essential for planning the proper treatment of TMD.

There were several limitations of the study. The baseline knowledge of the students was not evaluated before the courses. The traditional and online courses were held during different years although they all were at the third-year stage in their education. For the group size, an ad hoc effect size and sample size calculations were done and the sample size was sufficient for the analyses. However, although different student groups attended the traditional and online courses, this could also be considered a strength because they had no previous experiences about the course. All the third-year undergraduate dental students in two consecutive years were included. The fact that students in the online course had access to additional teaching materials in relation to the traditional course is also a limitation. The reliability of the questionnaires of self-assessed ability and satisfaction was tested and shown to be good. These questionnaires have been used earlier and were adopted from the study by Vilanova et al. who also evaluated the learning outcome of DC/TMD Axis I protocol, that is, compared the difference in diagnostic reliability between self-instructed examiners and examiners participating in DC/TMD course [14].

The present study results are based on undergraduate students’ self-evaluation of ability and satisfaction to perform the DC/TMD Axis I protocol. However, it would be necessary to evaluate whether these learning methods result in consistent diagnoses derived by a reference standard examiner. This comparison may better indicate the actual learning and could be an issue of future studies.

## Conclusion

In general, it could be argued that becoming acquainted with the DC/TMD protocol by only the written material is probably inadequate to perform the DC/TMD clinical examination and diagnosis with self-confidence. However, as shown in the present study, the web-based online learning course can promote learning as effectively as traditional lectures. As the online learning course received better results in measurements, identifying referred pain and statement of diagnostics in self-assessed ability and satisfaction before hands-on practice, it therefore could be an applicable method for education of the DC/TMD Axis I protocol in undergraduate dental education. The online learning course targeting on graduated dentists can be considered as a good option for online learning of the DC/TMD protocol also before the 2-day continuing education course.

None of the authors have a conflict of interest to disclose.

The participants of this study did not give written consent for their data to be shared publicly.
